# The Antioxidant Properties, Metabolism, Application and Mechanism of Ferulic Acid in Medicine, Food, Cosmetics, Livestock and Poultry

**DOI:** 10.3390/antiox13070853

**Published:** 2024-07-16

**Authors:** Mengli Zheng, Yating Liu, Guanfeng Zhang, Zhikang Yang, Weiwei Xu, Qinghua Chen

**Affiliations:** College of Animal Science and Technology, Hunan Agricultural University, Changsha 410128, China

**Keywords:** ferulic acid, ROS, antioxidant and anti-inflammatory, structure, application

## Abstract

Ferulic acid is a ubiquitous ingredient in cereals, vegetables, fruits and Chinese herbal medicines. Due to the ferulic phenolic nucleus coupled to an extended side chain, it readily forms a resonant-stable phenoxy radical, which explains its potent antioxidant potential. In addition, it also plays an important role in anti-cancer, pro-angiogenesis, anti-thrombosis, neuroprotection, food preservation, anti-aging, and improving the antioxidant performance of livestock and poultry. This review provides a comprehensive summary of the structure, mechanism of antioxidation, application status, molecular mechanism of pharmacological activity, existing problems, and application prospects of ferulic acid and its derivatives. The aim is to establish a theoretical foundation for the utilization of ferulic acid in medicine, food, cosmetics, livestock, and poultry.

## 1. Introduction

Ferulic acid (FA), also known as 4-hydroxy-3 methoxycinnamic acid, is an extremely abundant and almost ubiquitous phytochemical phenolic derivative of cinnamic acid that interacts with polysaccharides and oligosaccharides, and polyamine lipids. There is evidence that FA is widely present in plants (wheat, corn seeds), vegetables (lettuce), fruits (citrus, grapes) and Chinese herbal medicines (reed roots, angelica, etc.). However, the content of FA varies from one plant to another, depending on the type of plant [1]. The highest polyphenol content was found in maize grains (6056.9 mg/kg or 15.55 μmol/g) [2]. Taking corn seeds as an example, the presence of hydroxycinnamic acid and FA in the cell wall and embryo of corn aleurone was localized by focusing microscopy [3]. In addition, a previous study revealed that the content of FA in the corn anticline cell wall was twice as high as that in the pericarp cell wall [3].

The highest antioxidant capacity of cinnamic acid and its derivatives can be seen in the para-hydroxyl group [4]. The π-type delocalization of unpaired electrons on aromatic rings, double bonds, and o atoms is the key to the stability of free radicals. The ortho-dihydroxy substitution in the benzene ring positively affects the neutralization ability of free radicals, making caffeic acid an antioxidant of hydroxycinnamic acid [4]. Previously, chemical synthesis and structural confirmation of FA by spectroscopy described the presence of unsaturated side chains, as well as *cis*- and *trans*-isomer forms [5], and trans-FA with pharmacological functions was *trans*-FA. It had strong absorption in the ultraviolet range, with maximum absorption values of 284 nm and 307 nm in aqueous solutions at pH 6.0. It also exhibited intense fluorescence. In FA, resonant-stable phenoxy radicals were responsible for its potent antioxidant activity [6]. It catalyzed stable phenoxy radical formation when absorbing UV light, which provided strength for FA to terminate the radical chain reaction [7]. In addition, FA contributed antioxidant effects by providing hydrogen gas to free radicals with phenolic hydroxyl groups [6]. Also, FA had a reactive oxygen-generating function, which was similar to that of superoxide dismutase (SOD), an enzyme which protected organisms against the toxicity of reactive oxygen species.

In addition to being a free radical scavenger, FA is an inhibitor of the enzyme that catalyzes the production of free radicals, and is also considered to be an enzyme activity enhancer of the scavenger [8]. From the mechanism analysis, the antioxidant effect of FA mainly includes inhibiting the formation of reactive oxygen species (ROS) or nitrogen and neutralizing free radicals (forming stable phenoxy radicals through the reaction between free radical molecules and antioxidant molecules), which could inhibit the complex reaction cascade generated by free radicals. NADPH oxidase, xanthine oxidase, mitochondrial respiratory chain, lipoxygenase and nitric oxide synthase could be used as a variety of sources of ROS in cells [9]. An in vitro study confirmed that FA inhibited hydrogen peroxide (H_2_O_2_)-induced rat vascular smooth muscle cell injury by reducing malonydialdehyde (MDA) content, increasing SOD activity and Glutathione (GSH) content [10]. Mechanistically, FA pretreatment reduced ROS production by inhibiting NADPH oxidase expression, down-regulating the MAPK and AKT pathways [10]. Furthermore, FA could be used as a hydrogen donor to directly provide atoms for free radicals to protect cell membrane lipid acids from free radical oxidation. In addition, FA could chelate Cu (II) or Fe (II) metal ions, thereby preventing the formation of toxic hydroxyl radicals, leading to cell membrane peroxidation. Compared with other polyphenols, the pro-oxidation activity of FA was both similar and different. The pro-oxidation activity of polyphenols was produced by the following series of reactions to form OH radicals [11]. It was found that in the presence of Cu (II), compounds with o-dihydroxy (such as caffeic acid) and 4-hydroxy-3-methoxy (such as FA) had higher pro-apoptotic activity (preventing tumor cell survival and proliferation). Mechanistically, o-dihydroxyl was responsible for chelating Cu (II) ions and generating a CA-Cu (II) complex. Once the complex is formed, the second electron is transferred to O_2_, leading to the formation of O_2_^•−^ and the final product o-quinone, which damages DNA by forming covalent adducts or producing reactive oxygen species. In addition, a study showed that FA did not have the ability to chelate Cu (II), but it could induce DNA damage, confirming that FA could reduce Cu (II) to Cu (I) through intramolecular electron transfer [12].

As FA contains a variety of reactive groups, its molecular structure is easily modified. FA derivatives have the characteristics of high activity and low toxic effects. FA derivatives could be modified to obtain a variety of FA derivatives, which increases the application scope for FA. FA could also react with phenols or alcohols, sodium hydroxide, dibromoalkanes, and amino acids to obtain a series of derivatives such as esters, ether derivatives, feruloates, and amide derivatives. A number of FA derivatives are known, including FA piperazine, FA acerolate, ferulic acid 7-hydroxyisoflavone, ferulic acid glyceride, isoferulic acid, and so on [13,14,15]. Chinese physicians have been using piperazine ferulate to treat nephritis and nephrotic syndrome among other types of glomerular diseases [16]. Fish oil stored at high temperatures could be effectively delayed by 7-hydroxyisoflavone ferulate compared to FA [14].

**Figure 1:**
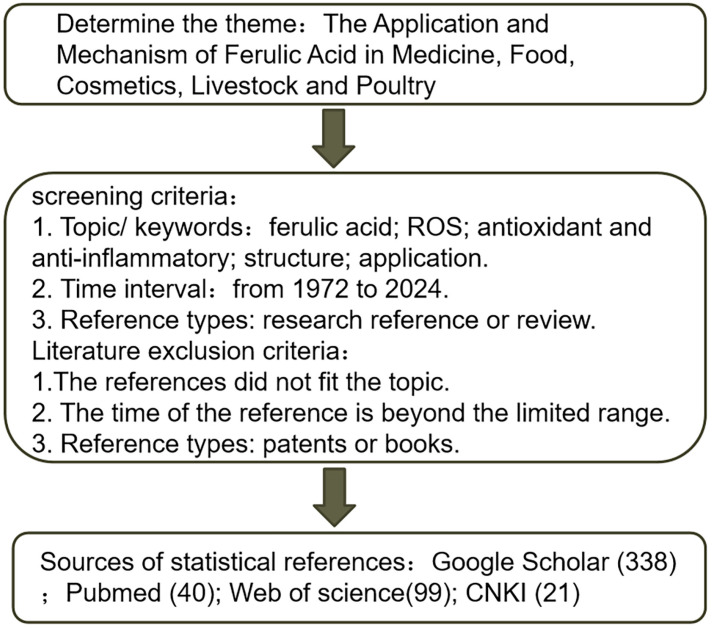
Reference sources and screening criteria.

Based on the antioxidant, antibacterial, anti-cancer, and fresh-keeping effects of FA, FA and its derivatives are widely used in the food industry, cosmetics industry, pharmaceutical industry, and feed industry (the sources and selection criteria for the references in our review are shown in Figure 1). However, the utilization rate of FA has not reached the expected level due to the poor water solubility of FA and the relatively poor ability to penetrate biological barriers. This article provides a theoretical basis for the promotion and efficient utilization of FA and its derivatives by reviewing their structure, application status, molecular mechanism of pharmacological activity, existing problems, and potential applications.

## 2. The source of FA and the Structure of FA in Lignocellulose

Many FAs are present in lignocellulose, especially in wheat, corn seeds, and Chinese herbal medicines such as reed root, angelica, and wild ferula resources (Table 1) [17,18,19,20,21].

**Figure 2:**
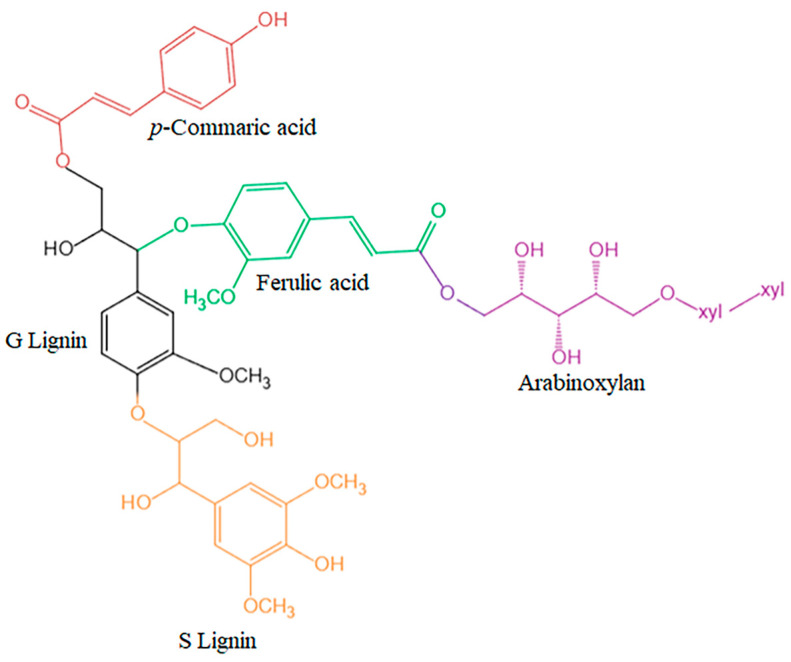
Schematic diagram of the connection of lignin phenolic carbohydrate complexes in the cell wall.

In lignocellulose, FA is often linked to ether bonds to arabinoxylan or lignin, and ester bonds to *β*-*p*-coumaric acid (Figure 2). Plant cell walls have antimicrobial properties due to the complex structure of FA intertwined with lignin, arabinoxylan, and lignin. FA has structural components of 3-methoxy and 4-hydroxy groups on the benzene ring, as well as carboxylic acid groups. These components either stabilize the obtained phenoxy radical intermediates, or even inhibit free radical chain reactions [6]. In addition, this carboxylic acid group could act as an anchor for ferulic acid, enabling it to bind to lipid bilayers and prevent lipid peroxidation.

## 3. The Molecular Basis for the Antioxidant Properties of FA and Its Derivatives

Exploring the mechanism of the antioxidant activity of phenolic acids and their derivatives has always been a hot topic for scientists. Regarding FA, a phenolic acid, it has been reported that the main antioxidant activity of FA and its derivatives (include 2-hydroxycinnamic acid, 3-hydroxycinnamic acid, *p*-coumaric, caffeic acid, etc.) has been theoretically evaluated through four mechanisms of action [22,23], such as hydrogen atom transfer (HAT) (ArOH + X^•^→ ArO^•^ + XH), single-electron transfer and proton transfer (SPL-ET) (ArOH → ArO^−^ + H^+^; ArO^−^ + X^•^ + H^+^ → ArO^•^ + XH), sequential proton loss electron transfer (SPL-ET) (ArOH → ArO^−^ + H^+^; ArO^−^ + X^•^ + H^+^ → ArO^•^ + XH), and an excessive metal-chelating agent (H_2_O_2_ + M^n+^ → HO^−^ + HO^•^ + M^(n+1)+^; ArOH → ArO^−^ + H^+^). The HAT theory holds that when the hydrogen atom is transferred from the polyphenol to another molecule, the coordination of the nucleus changes, which leads to the occurrence and development of the reaction. HAT is a one-step reaction related to OH bond dissociation enthalpy (BDE), while SET-PT and SPL-ET are two-step reactions. Generally, HAT is related to ionization potential (IP) and proton dissociation enthalpy, and SET-PT and SPL-ET are related to proton affinity (PA) and electron transfer enthalpy [24,25]. The transition metal-chelating agent theory holds that for polyphenols, each molecule that could be dissociated into anions and protons has the ability to chelate heavy metals, and metal chelation often occurs with the presence of deprotonated hydroxyl groups in polyphenols [26].

Indeed, the HAT mechanism by which FA and its other phenolic acid derivatives exert antioxidant properties is similar. Phenolic carboxylic acid antioxidants do not act as free radical scavengers alone in the body but form a complex antioxidant network together with the co-antioxidant GSH (glutathione). The differences in the antioxidant effects of different phenolic carboxylic acids could be evaluated using differential scanning calorimetry to assess their free radical scavenging ability. A previous study used the induction period (IP) method to investigate the effects of caffeic acid, *p*-coumaric acid, FA, and chlorogenic acid with or without 2-mercaptoethanol (ME) on the polymerization of methyl methacrylate (MMA) (to evaluate the free radical scavenging activity of ferulic acid and related compounds under almost anaerobic conditions) [27]. The results showed that the stoichiometric factors (*n*, free radical captured by 1 mole of antioxidant) of caffeic acid, ferulic acid, *p*-coumaric acid, and chlorogenic acid were 2.4, 1.8, 1.7, and 0.9, respectively, during free radical scavenging on PhCOO^•^, while the corresponding values for R^•^ free radical scavenging were 1.3, 1.2, 1.0, and 0.8, respectively [27]. This suggested that the free radical scavenging ability of ferulic acid was lower than that of caffeic acid, but higher than that of coumaric acid and chlorogenic acid.

The structure–activity relationship of ferulic acid and its related compounds is indeed a key point that we ignore. There are many kinds of phenolic acids, and it is also a hot topic to compare the antioxidant properties of different phenolic acids. Taking five cinnamic acids as examples, such as FA, caffeic acid, *o*-hydroxycoumaric acid, *p*-hydroxycoumaric acid, *m*-hydroxycoumaric acid and cinnamic acid, the effect of phenolic acid structure on antioxidant activity was revealed according to the antioxidant results and chemical structure. And the description of this part has been added to the revised draft.

A previous study found that different concentrations of cinnamic acid were added to the luminescence system, and the clearance rate of cinnamic acid to O_2_^−^ was reflected by monitoring the change in A560 value [28]. And the result showed that in the concentration range of 6.88 × 10^−7^ ~ 4.40 × 10^−5^ mol/L, the A560 value almost did not change with the increase in cinnamic acid concentration, indicating that cinnamic acid had little effect on the reduction of nitrotetrazolium blue to formazan by O_2_^−^ in this reaction system, revealing that cinnamic acid had no scavenging effect on O_2_^−^. Similarly, the half-maximal inhibitory concentration (IC50) values of caffeic acid, ferulic acid, *p*-hydroxycoumaric acid, *o*-hydroxycoumaric acid and *m*-hydroxycoumaric acid to O_2_^−^ were 19.64 × 10^−7^ mol/L, 92.43 × 10^−7^ mol/L, 270.7 × 10^−7^ mol/L, 349.89 × 10^−7^ mol/L and 480.9 × 10^−7^ mol/L, respectively [29]. It can be seen that the order of O_2_^−^ scavenging ability of these five phenolic acids from strong to weak is caffeic acid, ferulic acid, *p*-hydroxycoumaric acid, *o*-hydroxycoumaric acid and *m*-hydroxycoumaric acid.

The bond dissociation energy (BDE) is a thermodynamic parameter used to measure the difficulty of phenolic hydroxyl groups providing reducing hydrogen atoms to free radical molecules, and it is also an important parameter to characterize the antioxidant mechanism of HAT. The compounds with the lowest BDE_OH_ value had higher antioxidant activity. It was found that the gas phase BDE_OH_ values of molecules with active OH at ortho or para positions were lower than those of phenol, and the order was as follows: *p*-hydroxycoumaric acid > *o*-hydroxycoumaric acid > FA > caffeic acid. The presence of additional functional groups at the ortho position is beneficial to improve the antioxidant properties and reduce the BDE_OH_ in vacuum and polar media. *O*-hydroxylation (caffeic acid) is more favorable than *o*-methoxylation (FA). The antioxidant capacity of phenolic carboxylic acid antioxidants was studied by the B3LYP/6-31G (d,p) method in the Gauss 98 package. The BDE_OH_ of caffeic acid (327.34 KJ/mol) is about 12 KJ/mol lower than that of FA (339.04 KJ/mol), which is due to the formation of intramolecular hydrogen bonds between the 1 and 2 positions of caffeic acid, so the antioxidant activity of caffeic acid is stronger than that of ferulic acid [29]. In addition, when the substituent is located at the ortho and para positions of the phenolic hydroxyl group, the electron cloud distribution on the benzene ring is symmetrical, which makes the formed phenoxy radical more stable, so that the antioxidant activity of the ortho and para substituents of the phenolic hydroxyl group is greater than that of the meta position of the phenolic hydroxyl group (that is, the antioxidant activity of *p*-hydroxycoumaric acid and *o*-hydroxycoumaric acid is stronger than that of *m*-hydroxycoumaric acid) [30,31]. In summary, for ferulic acid and its derivatives (or phenolic acids), phenolic compounds with more electron-donating groups on the benzene ring and with the ability to form intramolecular hydrogen bonds have strong antioxidant activity.

## 4. Metabolic Process of FA

### 4.1. The Metabolic Process of FA in the Animal Organism

The bioavailability and fate of phenolic acids in the digestive process have been widely studied. With the order of food from the stomach-small intestine-large intestine, the bioavailable free phenolic acids in the stomach might bind to glucuronic acid and are then absorbed by the brush-like marginal cells in the small intestine, while the bound phenolic acids continue to enter the large intestine, where they are further metabolized and degraded by microbial esterases and bacteria [32]. The results of in vitro simulated digestion experiments showed that the release of esterified FA from the barley matrix was only possible by enzymatic action, and mainly occurred during intestinal digestion rather than gastric digestion. Another possible explanation for the elevated levels of esterified FA after intestinal digestion is that FA interacts with and binds to pancrea α tic enzymes, including pancreatic amylase, trypsin, and lipase. Molecular docking studies revealed that FA could interact with α-amylase and α-glucosidase through hydrogen bonding at the active site. In detail, monohydrogen bonding with α-amylase occurs between the C3 and C4 carbonyl groups and the amino acid Gly 334, exhibiting competitive inhibition. Other amino acid residues away from the active site exhibit non-competitive inhibition through hydrophobic interactions or van der Waals forces with FA. It is worth noting that the competitive interaction formed a stronger, more stable interaction between FA and α-amylase. It is worth noting that the competitive interaction formed a stronger and more stable interaction between FA and α-amylase. Inhibition of α-amylase reduces the ability to digest substrate starch [33].

### 4.2. The Metabolic Utilization of FA by Bacteria and Fungi

A portion of FA is released from plant cells after decomposition by enzymes produced by microorganisms in the animal’s gastrointestinal tract or environment, and then it is absorbed or further catabolized by them [34]. A major component of microbial catabolism is the production of other organic molecules by adding or deleting side groups, or the incorporation of carbon from other phenolic acids into biomass and polymeric fatty acids [35,36].

**Figure 3:**
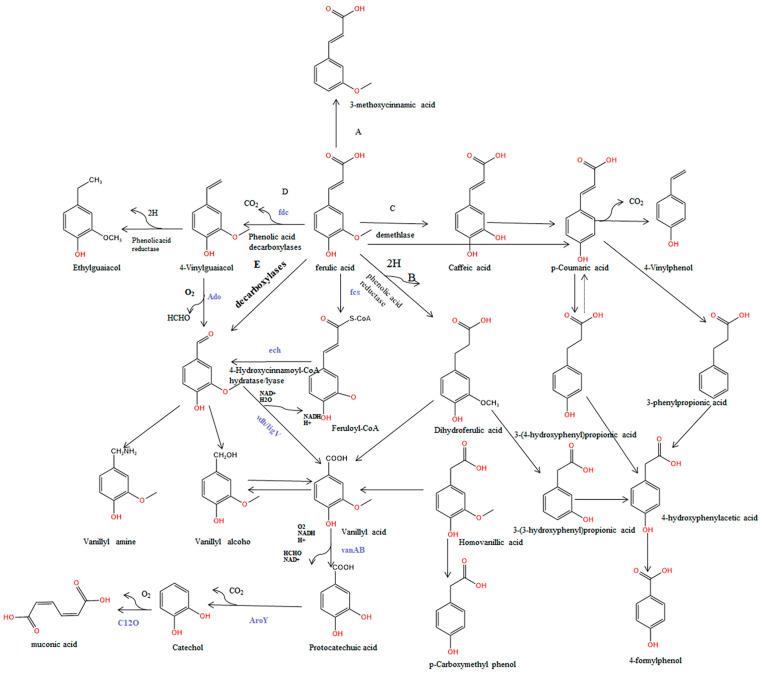
Metabolic processes of FA in bacteria or fungi. (A) Dihydroxylation; (B) restoration; (C) demethylation; (D) decarboxylation; (E) deacetylation. Fcs (this gene encodes feruloyl-CoA synthetase), ech (this gene encodes the enzyme enoyl-CoA hydratase/aldolase), and vdh (an enzyme that encodes vanilla dehydrogenase) refer to key genes in the coenzyme A-dependent β-free oxidation pathway. VanAB is a demethylase (the gene-encoding vanillic acid O-demethylase). The contents marked in purple are key genes in the metabolic pathway.

It was further noted that microorganisms were used in food, daily necessities, medicine, chemical and other fields to convert FA into high-value products, such as vanillin, lauryl alcohol, etc. [37]. Traditionally, FA undergoes non-oxidative decarboxylation [38], *β*-oxidation [39], demethylation [40], side-chain reduction [41], independent deacetylation by CoA and direct deacetylation [42,43]. There is evidence that *Pseudomonas putida* KT2440 produces protocatechuic acid using FA or vanillic acid [44]. Additionally, ferulic acid and *p*-coumaric acid can be synthesized using molecular biology methods in the *E. coli* system [45]. Research in bioengineering has revealed that vanillin is an aldehyde intermediate in the degradation pathway of ferulic acid. Several microorganisms, including *Pseudomonas* sp., *E. coli*, *Rhodococcus* sp., and *Bacillus subtilis* produce vanillin from ferulic acid [46,47,48,49]. By blocking vanillin catabolism, knocking out the vanillin dehydrogenase gene (vdh) is thought to increase vanillin production. FA also generates 4-vinylguaiacol through decarboxylation, which is further dehydrogenated and reduced to ethyllauryl alcohol [50]. By demethylating FA, caffeic acid could also be produced, and then *p*-coumaric acid can be synthesized, and 4-vinylphenol can be produced by decarboxylating *p*-coumaric acid [51]. On the basis of the prior literature, Figure 3 illustrates possible pathways for FA breakdown and metabolism in microorganisms.

### 4.3. Pharmacokinetics of FA, Including Absorption, Metabolism, Distribution, and Excretion

After oral ingestion, FA can be absorbed and utilized in the stomach, small intestine (jejunum and ileum), and colon (Figure 3). In fact, the colon is the main site of absorption of FA in the human body, and microbial esterases in the intestinal lumen hydrolyze esterified FA in food matrices to produce free FA. A previous study revealed that the absorption sites of free FA and fiber-bound FA are different. The former could be absorbed before reaching the ileum, but the latter must be degraded in the hindgut for further absorption and metabolism [52].

**Figure 4:**
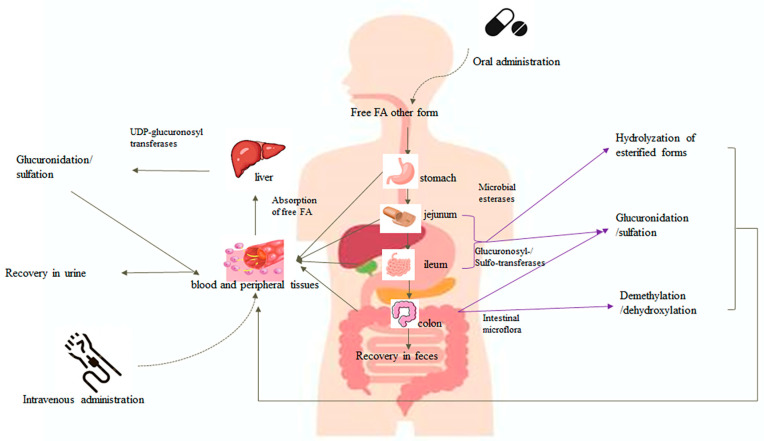
The digestion and absorption process of FA in the human body. The black arrow represents the metabolic pathway of FA, and the purple arrow represents the metabolic modification process.

The rat body delivers FA to the liver tissue via mutual circulation between the blood and peripheral tissues after intravenous injection of FA (Figure 4). A portion of free FA is absorbed during circulation and the majority of bound FA is further metabolized by the liver. One of the primary metabolites of FA is FA-sulfoglucuronide, and two other conjugated forms are FA-glucuronide and FA-sulfate. Under the action of sulfotransferase and UDP glucuronosyltransferase, FA is conjugated to FA-sulfoglucuronide, FA-glucuronic acid, and FA-sulfate [53,54]. The intestinal mucosa and kidney might be involved in this process. The binding of FA may be dose-dependent, because high FA levels may saturate the coupling enzyme, leading to the accumulation of free FA in plasma. In addition to coupling, metabolic reactions include double bond reduction, demethylation, dehydroxylation of C3 or C4, and methylation. The efflux of conjugates is also dependent on transporters. In addition to feruloylglycine, FA also produces vanillic acid, vanilloyl glycine, m-hydrophenyl propionic acid, dihydroferulic acid, feruloyl sulfate, and trans-feruloyl-4-O-β-D-glucuronide [52,55].

In humans, FA is excreted through urine and reaches a plateau 7~9 h after ingestion. There was an approximate 5% recovery of free FA in rat urine and an approximate 11–25% recovery of total FA in both free and conjugated forms (mainly feruloyl glucuronide) in human urine. Comparatively to total excretion, the percentage of sulfonated FA in urine reached 84%. Only 0.5–0.8% of ingested FA was found in the feces of rats, indicating that the absorption rate of FA was very efficient.

## 5. The Current Application Status and of FA and Its Derivatives

**Figure 5:**
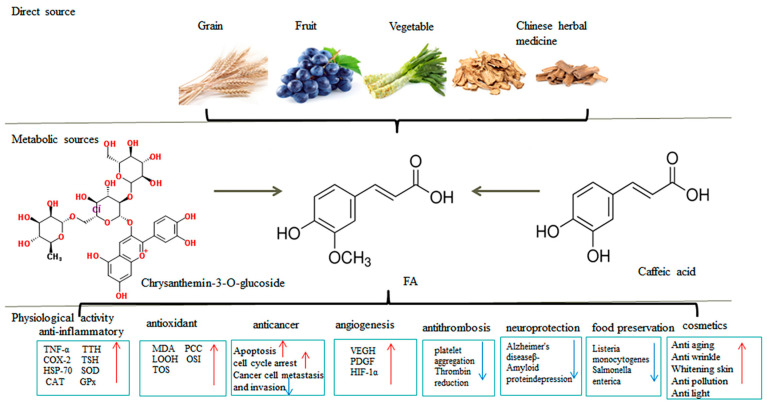
The source and physiological activity of FA. Note: The red arrow represents the promoting effect, and the blue arrow represents suppression or reduction effect.

Based on the great antioxidant effect of FA, FA and its derivatives are widely used in the food industry, cosmetics industry, pharmaceutical industry and feed industry. In the food industry, FA plays a variety of functions, including antioxidants, antibacterials, food preservatives, food glue, edible packaging films, lipid antioxidants, and sausage preservation [56,57,58] (Figure 5). Additionally, FA derivatives could improve lung endothelial infection and lung volume in humans [59]. FA is thought to inhibit melanism by antagonizing tyrosine because of their similar chemical structure.

Overexposure of human skin to ultraviolet radiation can cause skin damage such as UV-induced skin damage, skin photoaging (atrophy, pigmentation changes and wrinkles), sun damage, skin sensitivity and malignancy. In the cosmetics industry, FA could improve skin conditions, protect against ultraviolet radiation, delay aging, and inhibit melanin accumulation [60]. FA derivatives can be used for skin whitening care or freckle removal treatments, strongly inhibiting the formation of melanin and inhibiting tyrosinase activity [61]. Moreover, The incorporation of ferulic acid into a topical solution containing 15% L-ascorbic acid and 1% α-tocopherol improves the chemical stability of vitamins (C+E), manifested in an increase in the photoprotection of the skin from 4 to about 8 times when the sun simulates irradiating it [62].

Ferulic acid has weak hydrophilicity and lipophilicity, which limits its absorption and utilization by the skin, and also weakens its antioxidant properties. The poor water solubility of FA limits its use and the possibility of incorporating it at high concentrations into hydrophilic local formulations [63]. The emergence and progress of new delivery systems for active ingredients promoted the development of skin pharmaceuticals and cosmetics. Cyclodextrin (CD) is a cyclic oligosaccharide that could form a host–guest inclusion complex with guest active molecules, thereby improving the physical and chemical properties of such molecules. The active ingredient–CD inclusion complex has been described as an advantage of promoting skin penetration of active ingredients and improving anti-aging preparations, with unique biocompatibility characteristics. In addition, using α-CDs, β-CDs, γ-CDs, HP-β-CDs and HP-γ-CDs as skin carriers, nanomaterials that can be dispersed in aqueous and oil phases were prepared for cosmetic applications. Due to the poor water solubility of trans-ferulic acid, the researchers evaluated the different carbon dot behaviors of trans-ferulic acid. The results showed that the apparent water solubility of trans-ferulic acid-α-CD, trans-ferulic acid-Me-β-CD, trans-ferulic acid-HP-β-CD and trans-ferulic acid-HP-γ-CD increased by 5.0-fold, 4.8-fold, 4.5-fold and 8.3-fold, respectively, compared with free trans-ferulic acid [64]. It can be seen from the results that the apparent water solubility of trans-ferulic acid-HP-γ-CD increased the most. Furthermore, the study revealed that the trans-ferulic acid-HP-γ-CD inclusion contained the largest sunscreen coefficient (SPF) and UVA protection factor. Although CD has the advantages of increasing the solubility, stability, permeability and retention of active ingredients, there is still a lack of clinical research evidence for the application of CD skin medicine and cosmetics and compatibility with other ingredients in the formulation. Currently, researchers often use choline salts of amino acids as green excipients to improve the solubility and content of ferulic acid in formulations. Research has shown that novel FA derivatives synthesized using glycine (GPr [FA]), L-leucine (LPr [FA]), and L-proline (PPr [FA]) as three amino acid propyl esters can improve the solubility and permeability of ferulic acid, making them an interesting alternative for dermatology and cosmetic preparations [65]. The absorption and penetration of compounds in the skin depend on their physical and chemical properties, especially their lipophilicity. Due to the low bioavailability of FA, its derivatives can be considered as substitutes. Throughout the chemical structure of FA, it contains phenyl, hydroxyl, and carboxyl groups, ethylene bonds, and benzene rings. Therefore, the structural characteristics of FA make it the optimal substrate for synthesizing various derivatives and increasing skin permeability.

In the pharmaceutical industry, FA has antibacterial and anti-inflammatory, analgesic and antithrombotic, antioxidant, and anti-tumor effects; it improves human immune function and reduces blood concentration [66]. FA has antimicrobial activity, such as antibacterial effect against the pathogenic bacteria *Escherichia coli* O157: H7 ATCC 43888 and *Listeria monocytogenes* ATCC 7644 [67], as well as *Staphylococcus aureus* [68]. The anti-cancer activity of natural FA stems primarily from its ability to inhibit reactive oxygen species, which protect cellular components such as DNA, peptides, and lipids from oxidative damage. In addition, the activity of FA is due to the regulatory effect of FA on intracellular signaling pathways, proliferation, apoptosis, and metastasis. Consistent with FA, FA derivatives also have antithrombosis, anti-inflammatory, anti-cancer, regulating human immune function, treating cardiovascular and cerebrovascular diseases, protecting the brain, and alleviating cerebral infarction [69].

In the feed industry, the higher the content of FA in the feed, the more conducive it is to the digestion and utilization of plant-based feed. It also improves the anti-inflammatory and antioxidant effects in animals. Moreover, FA and other hydroxycinnamic acids, including caffeic acid and *p*-coumaric acid, all inhibit the increase in *Listeria monocytogenes*, which is beneficial for the preservation and storage of food [70].

The addition of ferulic esterase to animal feed, or the treatment of animal feed with ferulic esterase-producing microorganisms, can improve the digestibility and antioxidant activity of the feed, and promote the growth and development of animals [71]. In lignocellulose, the formation of ester bonds between FA and lignin restricts fiber digestibility, but the hydrolysis of these ester bonds by ferulic acid esterase is expected to improve rumen fiber degradation, energy supply and animal productivity [71,72]. Adding 250 or 500 mg/kg of FA to the feed of heifers could increase the average daily weight gain by 21%, and the weight of hot carcass weight and cold carcass weight were increased by 1.8% and 1.6%, respectively [73]. In cold environments, adding 80 mg/kg of ferulic acid to the diet of male lambs could increase the concentration of serum total protein and albumin, as well as the activity of plasma glutathione peroxidase and catalase, and reduce the content of malondialdehyde [74]. The results indicated that supplementing lambs with ferulic acid could reduce oxidative stress in cold environments, thereby improving their growth performance. A study of ferulic acid in poultry found that soaking chicken embryonic eggs in FA solution enhanced liver antioxidant capacity by upregulating the expression of Nrf2 Keap1 signaling pathway-related genes [75]. In addition, a study revealed that supplementing with FA in broiler chickens could alleviate LPS-induced intestinal injury, increase the level of secreted immunoglobulin A (sIgA), and regulate the composition of the ileal microbiota in broiler chickens attacked by LPS. Adding 0.1%, 0.2%, or 0.3% ferulic acid preparations to the feed of chicks could increase average daily weight gain, increase the number of lactic acid bacteria, and reduce the number of *Escherichia coli* [76]. In summary, adding ferulic acid to the diet could improve the production performance, blood characteristics, and gut microbiota of broiler chickens.

## 6. Molecular Mechanism of Pharmacological Activity of FA and Its Derivatives

### 6.1. Atherogenesis Was Improved by FA

**Figure 6:**
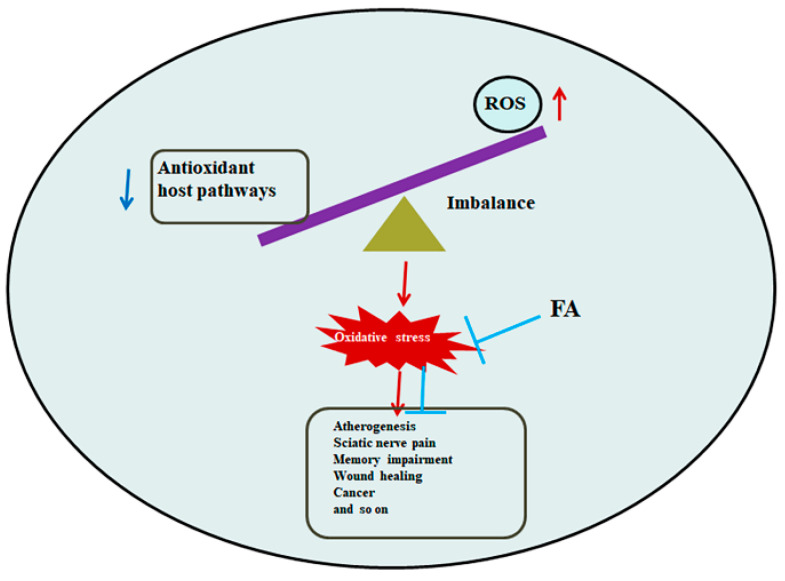
FA regulates the imbalance caused by oxidative stress. Note: The red arrow represents the promoting effect, and the blue arrow represents the inhibiting or reduction effect.

Atherosclerosis is the pathological basis of cardiovascular and cerebrovascular diseases and the leading cause of disease and disability in the world [77]. Oxidative stress, caused by the production of excess reactive oxygen species, has become a critical and ultimately common mechanism in atherosclerosis (Figure 6). The excessive proliferation and migration of vascular smooth muscle cells (VSMCs) are closely related to the occurrence and development of Atherosclerosis [78]. Cyclophilin A was found to be a 20 kD chaperone protein secreted by VSMCs in response to ROS, stimulating VSMC proliferation and inflammatory cell migration in vitro and in vivo [79]. In the process of Atherosclerosis development, it is easy to cause lipid deposition and thickening of the intima of the large and middle arteries, and then gradually form plaques, which cause narrowing of the lumen, and plaque rupture will lead to the formation of thrombosis, causing arterial blood supply disorders. A previous study found that FA inhibited the increase in adhesion factors in endothelial cells, blocked the free radical chain reaction, decreased the content of inflammatory factors, and reduced the increase in foam cells [80]. In addition, FA could also ameliorate atherosclerotic injury by modulating gut microbiota and lipid metabolism [81]. FA inhibited the proliferation of vascular smooth muscle cells through the NO/p21 signaling pathway, thereby alleviating atherosclerotic plaques in ApoE^−/−^ mice [82]. A study revealed that FA significantly alleviated atherosclerosis and improved lipid metabolism in ApoE^−/−^ mice, including up-regulation of AMPKα phosphorylation and down-regulation of SREBP1 and ACC1 expression [83]. In addition, FA induced significant structural changes in gut microbiota and fecal metabolites in atherosclerotic mice, and specifically reduced the relative abundances of *Fimicutes*, *Erysipelotrichaceae*, and *Ileibacterium*, which were positively correlated with blood lipid levels in atherosclerotic mice [79,84]. These results suggested that FA, as an antioxidant, could significantly ameliorate atherosclerotic injury, which might be partly through the regulation of gut microbiota and lipid metabolism through the AMPKα/SREBP1/ACC1 pathway, or inhibition of vascular smooth muscle cell proliferation through the NO/p21 signaling pathway.

### 6.2. FA Protected Nerves and Improved Memory Efficacy

FA has potential advantages in the treatment of sciatica. Previously, many references reported that ferulic acid has the effects of protecting nerves and improving memory (Table 2) [85,86,87,88]. The mechanisms of high-fat diet-induced cognitive impairment are complex and include inflammation, peripheral nervous system and brain insulin resistance, impaired glucose metabolism, increased oxidative stress, and mitochondrial dysfunction [88]. Insulin receptor substrate 1 (IRS1)/phosphatidylinositol 3-kinase (PI3K)/protein kinase B (AKT)/glycogen synthase kinase-3β (GSK3-β) is the most important signaling pathway for regulating blood glucose levels [87]. At the cellular level, FA increased the survival rate of palmitic acid (PA)-treated HT22 cells, inhibited apoptosis, and reduced oxidative stress through the IRS1/PI3K/AKT/GSK3β signaling pathway [87]. In addition, FA reversed the low expression of Nrf2 and Gpx4 proteins in mice caused by high-fat diets [88,89]. These results suggest that FA may exert antioxidant and anti-apoptotic effects through the IRS1/PI3K/AKT/GSK-3β pathway, thereby delaying cognitive deficits caused by hyperlipidemia. Furthermore, it was found that FA exerts a neuroprotective effect on radiation-induced nerve injury by targeting the NLRP3 inflammasome to enhance learning and memory ability and improve the pathological changes of hippocampal tissue in irradiated mice.

### 6.3. Wound Healing Was Facilitated by FA

Chronic hyperglycemia leads to an overproduction of free radicals, which can lead to oxidative stress, which can impair wound healing. The study on diabetic rats found that wound epithelialization was faster in the treatment of FA-loaded polymer nanoparticle dispersions (oral administration) and FA-loaded polymer nanoparticle-based hydrogels (topical administration) compared to diabetic wound controls [90]. Moreover, in vitro scratch wound assays demonstrated that the FA released by cross-linked alginate dialdehyde and gelatin enhanced the mobility of human dermal fibroblasts [91].

### 6.4. Cancer Suppressant

ROS alters the function of lipids, nucleic acids, and proteins by destroying them. Oxidative stress occurs when the balance between ROS production and antioxidant defense is disturbed, thus it often causes many diseases, including cancers such as breast cancer, lung cancer, etc. FA induces apoptosis and cell-cycle arrest (human breast cancer, non-small cell lung cancer, osteosarcoma), anti-angiogenesis of FA (human umbilical vein endothelial cells), inhibition of metastasis and invasion (colorectal cancer cell line), anti-inflammatory mechanism (which could reduce the production of macrophage inflammatory protein-2 (MIP-2)) [92]. FA-triggered apoptosis and autophagic cell death depend on the production of intracellular reactive oxygen species in various cancer cell lines. The potential apoptotic effect of FA is mediated by altered expression of precaspase-3, precaspase-8, precaspase-9, poly (ADP) ribose polymerase, Bcl-2, and Bax. It blocks the activation of classical SMAD and non-canonical extracellular signal-regulated kinase/Akt (protein kinase B) pathways in various cancer cells [93]. Furthermore, mice exhibiting lung injury and fibrosis were detected in an animal model of silica-induced pulmonary fibrosis (PF), which alleviated the accumulation of inflammatory cells in bronchoalveolar lavage fluid in mice, and demonstrated that FA stopped the progression of PF and prevented epithelial-mesenchymal transition (EMT) in a dose-dependent manner by improving the expression of fibrotic proteins (including collagen I, TGF-β, p-smad2/3) [94]. Combination treatment with thymoquinone and FA reduced the proliferation of the breast cancer cell line MDA-MB 231 cells [95].

### 6.5. Other Functions

FA could protect the liver [96], improve diabetes [97], etc. Due to its aromatic phenol ring, FA is able to stabilize and delocalize unpaired electrons, thereby acting as a free radical scavenger [98]. Balasubashini et al. [99] found that oral administration of 10 mg/kg FA for 45 days reduced streptozotocin (STZ)-induced hepatic oxidative stress in Wistar rats by increasing the activity of antioxidant enzymes such as glutathione peroxidase (GPx), SOD, and catalase (CAT). Narasimhan et al. [100] found that FA effectively enhanced glycogen levels by increasing the activity of hepatic GK enzymes and decreasing the activity of G6Pase and PEPCK. These results suggested that FA might regulate glucose homeostasis by ameliorating disorders of hepatic glucose metabolism.

## 7. The Problems and Application Prospects of FA

At present, there are still many problems in the application process of FA. For example, FA has relatively poor solubility in water and weak ability to penetrate biological barriers. Secondly, there is a lack of research on improving the bioavailability of monomeric components and specifically targeting them to tumor tissues through chemical modifications. In addition, there are significant differences in the doses used in FA anti-tumor studies, which are still in the exploratory stage and have not yet formed a unified standard. In order to widely expand the application of FA, a carrier can be developed to selectively deliver drugs to certain parts of the body, identify mutant cells, control drug release, determine safe and effective FA doses, and develop more effective drug regimens to serve patients more effectively.

## 8. Conclusions and Prospect

This article discusses the origin, structure, application and mechanism of FA. FA protects skin, inhibits cancer, improves memory disorders and other diseases by scavenging free radicals and reducing oxidative damage, which strongly supports the treatment of oxidative stress-related diseases. Furthermore, the article affirms that FA has a broad application market and could be used to develop new drugs, skin care products and food preservation.

## Figures and Tables

**Table 1:** The source and contents of FA.

Sources	Contents	References
wheat	0.117~0.212%	[17]
corn	0.0165	[18]
reed root	0.069%	[19]
angelica	0.05%	[20]
wild ferula *sinkiangensis* KM Shen	0.07%	[21]

**Table 2:** The mechanism of FA in protecting nerves and improving memory.

In Vivo or In Vitro Experiments	Treatment Methods or Dosage	Results or Mechanism of Action	References
In vivo experiment	A rat model of CCI pain was established with sciatic nerve ligation; 21 days of treatment with 100 mg/kg FA or 20 mg/kg methylcobalamin.	The levels of MDA, TNF-α, IL-β and IL-6 proteins in rat tissues were decreased.	[85]
In vitro experiment	LPS induces M1 polarization in GMI-R1 cells through the RhoA/Rock pathway.	FA could reduce the levels of inflammatory cytokines TRPA1 and TRPV1 through the RhoA/p38 MAPK pathway inhibiting peripheral sensitization in rats with chronic systolic injury, thereby relieving sciatica.	[86]
In vitro experiment	PA-treated HT22 cells.	FA increased the survival rate of PA-treated HT22 cells, inhibited cell apoptosis, and reduced oxidative stress through the IRS1/PI3K/AKT/GSK3β signaling pathway.	[87]
In vivo experiment	Mice, caused by high-fat diets.	FA reversed the low expression of Nrf2 and Gpx4 proteins in mice caused by high-fat diets.	[88]

Controlled cortical impact (CCI); malonydialdehyde (MDA); tumor necrosis factor-α (TNF-α); Interleukin-1 β (IL-β), IL-6, transient receptor potential A1(TRPA1), the transient receptor potential vanilloid (TRPV), ras homolog family member A (RhoA), p38 mitogen-activated protein kinase (p38 MAPK); palmitic acid (PA); Insulin Receptor Substrate-1 (IRS1); Phosphatidylinositide 3-kinase (PI3K); protein kinase B (AKT); glycogen synthase kinase 3β (GSK3β); nuclear factor erythroid 2-related factor 2 (Nrf2); Glutathione Peroxidase 4 (Gpx4).
